# Apolipoprotein E polymorphisms and female fertility in a transgenic mouse model of Alzheimer’s disease

**DOI:** 10.1038/s41598-024-66489-w

**Published:** 2024-07-10

**Authors:** Bani Medegan Fagla, Jason York, Amy Christensen, Cielo Dela Rosa, Deebika Balu, Christian J. Pike, Leon M. Tai, Irina A. Buhimschi

**Affiliations:** 1 Department of Obstetrics Gynecology, University of Illinois at Chicago College of Medicine, 820 S. Wood Street, Chicago, IL 60612 USA; 2 Department of Anatomy and Cell Biology, University of Illinois at Chicago College of Medicine, Chicago, IL 60612 USA; 3 Davis School of Gerontology, University of Southern California, Los Angeles, CA 90089 USA

**Keywords:** Experimental models of disease, Disease model, Alzheimer's disease, Reproductive biology

## Abstract

Apolipoprotein E (APOE) is a major cholesterol carrier responsible for lipid transport and injury repair in the brain. The human *APOE* gene (h-*APOE*) has 3 naturally occurring alleles: ε3, the common allele; ε4, which increases Alzheimer’s disease (AD) risk up to 15-fold; and ε2, the rare allele which protects against AD. Although *APOE4* has negative effects on neurocognition in old age, its persistence in the population suggests a survival advantage. We investigated the relationship between *APOE* genotypes and fertility in EFAD mice, a transgenic mouse model expressing h-*APOE*. We show that *APOE4* transgenic mice had the highest level of reproductive performance, followed by *APOE3* and *APOE2*. Intriguingly, *APOE3* pregnancies had more fetal resorptions and reduced fetal weights relative to *APOE4* pregnancies. In conclusion, *APOE* genotypes impact fertility and pregnancy outcomes in female mice, in concordance with findings in human populations. These mouse models may help elucidate how h-*APOE4* promotes reproductive fitness at the cost of AD in later life.

## Introduction

A*polipoprotein E* (*APOE*) is a polymorphic gene with three naturally occurring alleles in humans: ε2 (*APOE2)*, ε3 (*APOE3)*, and ε4 (*APOE4)*. Each of the alleles and their respective protein isoforms (APOE2, APOE3, and APOE4) have well-documented structural and functional differences that confer distinct associations between each genotype and a variety of health conditions. Notably, *APOE4* has been shown to increase the risk of several neurological and cerebrovascular disorders, including late-onset Alzheimer’s disease (AD) and cardiovascular diseases such as hypertension and atherosclerosis^1^. This has raised questions as to why the allele has been conserved throughout evolution at frequencies as high as 49% despite its association with phenotypically deleterious effects on health^2^.

According to evolutionary theory, for genetic polymorphisms to persist through natural selection, there must be beneficial effects from these mutations that grant humans a relative survival advantage. This phenomenon, known as “antagonistic pleiotropy”, can be applied to *APOE* genetic polymorphisms^3,4^. Indeed, despite its harmful effects later in life, the *APOE4* allele has been associated with increased immune protection in populations with a high infection burden^5,6^. There is also evidence that *APOE4* may have been advantageous in environments with limited food availability due to its ability to promote fat storage^7^. Importantly, it has been proposed that *APOE4* confers a reproductive advantage by increased fertility^5,8,9^. In a recent study, Trumble et al. reinforced this paradigm by demonstrating that *APOE4* was linked to increased number of children, earlier reproduction age, and shorter inter-birth interval in the Tsimane ethnic group of Bolivia^8^. This effect of *APOE* genotype on fertility has been postulated to result in superior survival and reproductive fitness for *APOE4* carriers. Therefore, identifying mechanisms that contribute to the effect of *APOE* genotype on reproduction may be important for understanding the role of *APOE* in the periphery and in brain function throughout the lifespan. In vivo transgenic models that express human *APOE* (h-*APOE*) have been developed to determine the impact of *APOE* genotype on neural function^10,11^. However, whether the observed differences in fertility among the *APOE* genotypes found in humans exist in these models is unknown. Establishing an animal model to study the effect of *APOE* genotypes on fertility will enable future studies focused on the underlying mechanisms of the reproductive phenotype effects on neurocognitive function during aging.

To that end, the goal of this study was to determine whether *APOE* genotype-associated effects on reproductive fitness can be recapitulated in animal models*.* To achieve this goal, we conducted a retrospective analysis of breeding records from three different animal colonies and evaluated intra-gestational status and outcomes in EFAD mice, a well-characterized APOE knock-in (KI) model expressing the different h-*APOE* (E) alleles in addition to familial AD (FAD) transgenes. Specifically, the EFAD mouse model was developed by crossing APOE-target replacement (APOE-TR) mice, a KI model expressing h-*APOE* instead of mouse *APOE,* with 5xFAD mice which co-express five FAD mutations that result in overproduction of Aβ^10,12,13^. As a result, EFAD mice are 5xFAD^+/−^/h-*APOE*^+*/*+^. The cross also results in EFAD non-carrier mice (EFAD−) which express h-*APOE* without the AD-associated transgenes (5xFAD^−/−^/h-*APOE*^+/+^). Thus, the functional effects of *APOE* can be determined in the presence (EFAD) and absence (EFAD−) of high Aβ pathology. EFAD mice were developed to reveal pathophysiological mechanisms associated with *APOE4* during aging and in AD, and have been extensively characterized in mechanistic and preclinical research^10^. Overall data has demonstrated that, in the EFAD model, *APOE4* carriers have greater neuroinflammation, neurovascular dysfunction, Aβ levels, neuron dysfunction, and cognitive deficits compared to *APOE3* carriers^10^. Furthermore, these effects are exacerbated in females, as found in humans^14–18^. Therefore, we considered EFAD mice an ideal APOE KI model to test the effects of *APOE* on female reproduction in vivo. We hypothesized that similar to epidemiologic findings in humans, h-*APOE4* carrier mice would have higher fertility compared to their *APOE3* and *APOE2* counterparts.

## Results

### Fertility in E2FAD, E3FAD, and E4FAD mice

**Figure 1:**
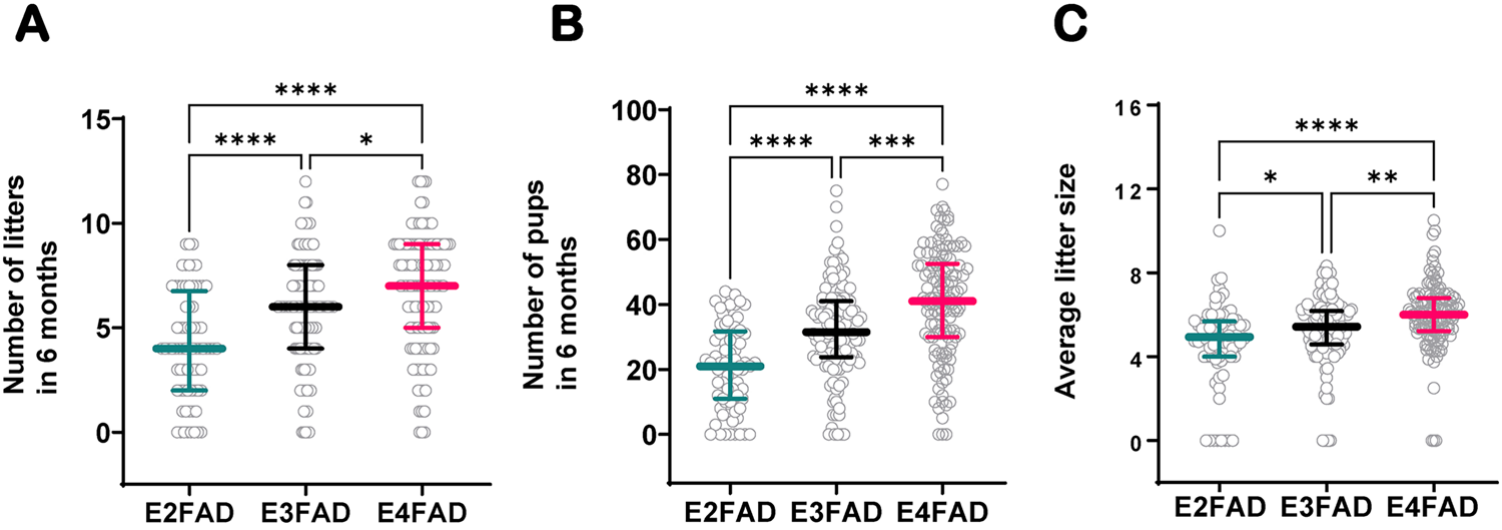
Fertility in colony I (EFAD). Number of litters (**A**) and number of pups (**B**) produced over a period of 6 months by E2FAD (n = 76), E3FAD (n = 130), and E4FAD (n = 125) breeding cages. Average litter size per breeding cage (**C**). Data is represented as scatterplots with median ± IQR. Statistical analysis: Kruskal–Wallis ANOVA on Ranks followed by multiple comparisons using Dunn’s post hoc test *p ≤ 0.05, **p ≤ 0.01, ***p ≤ 0.001, ****p ≤ 0.0001. In this colony, each breeding cage consists of trios of two females paired continuously with one male over the 6-month period.

To determine whether genotype-specific differences in fertility exist in mice expressing h-*APOE*, we first analyzed breeding records from two EFAD colonies labeled colony I and colony II. The characteristics and reproductive output of the colonies included in this analysis are presented in Tables 1 and 2 respectively. In colony I, which included EFAD mice carrying h-*APOE2* (E2FAD), h-*APOE3* (E3FAD), and h-*APOE4* (E4FAD), we analyzed a total of 76, 130, and 125 breeding cages, respectively. Out of the total number of breeding cages initially setup, 10.5% of E2FAD (8/68), 3.9% of E3FAD (5/130), and 2.4% of E4FAD (3/125) of breeding cages did not produce any litter (set of offspring in a single pregnancy) (χ^2^ = 7.24, p = 0.027) (Table 2). To assess fertility, we extracted the number of litters and number of pups (newborns < 3 days old) produced per breeding cage over a period of 6 months, as well as average litter size per breeding cage. Reproductive performance, as defined by these three metrics, was compared between genotypes. Our analysis revealed that reproductive performance decreased in a genotype-dependent manner (E4 > E3 > E2) (Fig. 1). Specifically, with a median value of 7.0 (5.0–9.0) litters, E4FAD mice had a significantly higher number of litters produced over 6 months than E3FAD (p = 0.028) and E2FAD (p < 0.0001) mice with a median of 6.0 (4.0–8.0) and 4.0 (2.0–6.8) respectively (Fig. 1A). The same trend was true for the total number of pups produced per breeding cage with a median of 41.0 (30.0–52.5) pups for E4FAD, 31.5 (23.8–41.0) for E3FAD and 21.0 (11.0–31.8) for E2FAD (p < 0.001) (Fig. 1B). Additionally, the average litter size was also significantly higher in E4FAD compared to E3FAD (p = 0.001) and E2FAD (p < 0.001) breeding cages (Fig. 1C). Finally, our analysis shows that E2FAD mice had the lowest reproductive performance of the three genotypes, with a significantly lower total number of litters and pups than E3FAD mice (p < 0.0001), as well as smaller litter size (p = 0.019) (Fig. 1A–C).

**Table 1:** Summary characteristics of the mouse colonies analyzed. Description of the colonies, including the principal investigator, mouse model genetic background, location, light and dark cycle, breeding scheme, and average age of females at first mating.

**Colony I**	
Principal investigator	Mary Jo LaDu, PhD^10,12,16,17,64^
Mouse model genetic background	EFAD (5xFAD^+/−^/APOE^+/+^)
Location of colony	Univ. of Illinois at Chicago, Chicago IL
Light–dark cycle	14:10
Breeding scheme	Trio (2F × 1M)
Age of females at initial mating (weeks)	
E2FAD (5xFAD^+/−^/APOE2^+/+^)	9.2
E3FAD (5xFAD^+/−^/APOE3^+/+^)	9.1
E4FAD (5xFAD^+/−^/APOE4^+/+^)	8.4
**Colony II**	
Principal investigator	Christian Pike, PhD^17,18,65–67^
Mouse model genetic background	EFAD (5xFAD^+/−^/APOE^+/+^)
Location of colony	Univ. of Southern California, San Diego CA
Light–dark cycle	12:12
Breeding scheme	Pair (1F × 1M)
Age of females at initial mating (weeks)	
E3FAD (5xFAD^+/−^/APOE3^+/+^)	11.4
E4FAD (5xFAD^+/−^/APOE4^+/+^)	13.2
**Colony III**	
Principal investigator	Leon Tai, PhD^10,11,13,68–70^
Mouse model genetic background	EFAD− (5xFAD^−/−^/APOE^+/+^)
Location of colony	University of Illinois at Chicago, Chicago IL
Light–dark cycle	14:10
Breeding scheme	Trio (2F × 1M)
Age of females at initial mating (weeks)	
E3FAD− (5xFAD^−/−^/APOE3^+/+^)	7.4
E4FAD− (5xFAD^−/−^/APOE4^+/+^)	6.7

**Table 2:** Reproductive output of each mouse colony analyzed. Total number of breeding cages or single pairings, total number of females, and proportion of non-productive cages for each animal colony in our study. Outcomes are shown for each *APOE* genotype represented in each colony.

Variables	*APOE2*	*APOE3*	*APOE4*
**Colony I**			
Total number of breeding cages (n)	76	130	125
Total number of females (n)	152	282	248
Number of non-productive cages (n)	8	5	3
Proportion of non-productive cages (%)	10.5	3.9	2.4
**Colony II**			
Total number of pairings (n)	–	286	230
Total number of females (n)	–	146	114
Number of non-productive cages (n)		88	35
Proportion of non-productive pairings (%)	–	30.8	15.2
**Colony III**			
Total number of breeding cages (n)	–	31	34
Total number of females (n)	–	62	68
Number of non-productive cages (n)		3	0
Proportion of non-productive cages (%)	–	9.6	0.0

**Figure 2:**
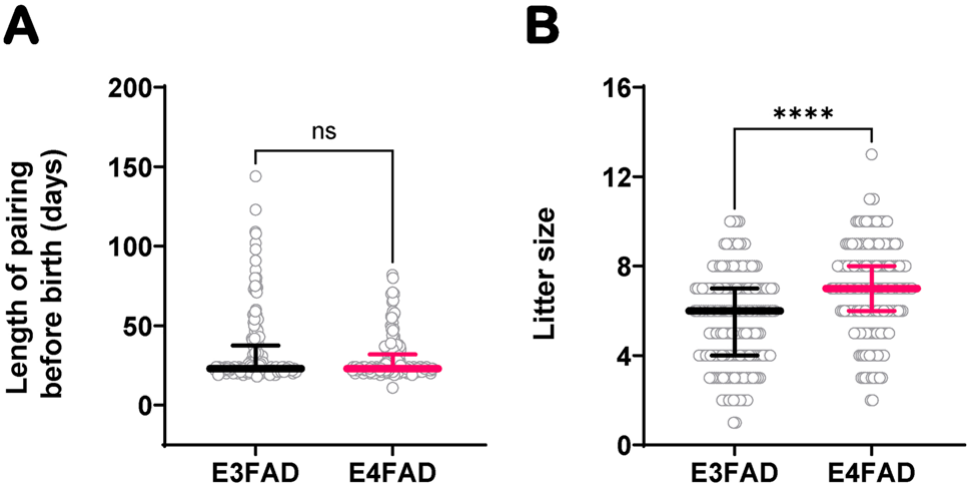
Fertility in colony II (EFAD). Litter size (**A**) and length of pairing with a male before birth of a litter (**B**) among E3FAD (n = 198) and E4FAD (n = 195) productive pairings. Data is represented as scatterplots with median ± IQR. The differences between each group were determined by Mann–Whitney Rank Sum test. ****p ≤ 0.0001. In this colony, breeding scheme consists of a female paired with a male until the birth of a litter. Each female is paired for 1 to 4 consecutive times with a single male. The male used varies between each pairing.

To exclude the possibility of a colony specific phenotype, we performed a similar assessment in colony II, a second colony of EFAD mice with different breeding protocols. In colony II, which had E3FAD and E4FAD breeders, but not E2FAD, we estimated the length each female was paired with a male before a live birth occurred and we extracted the median litter size for all litters produced per breeding female. We found that, while the length of pairing before birth of a litter did not significantly vary between genotypes (Fig. 2A), E3FAD females had significantly smaller litters than E4FAD females (p < 0.001) (Fig. 2B). Even though colony II followed a different breeding scheme, these results are concordant with the trend observed in colony I. Importantly, out of all pairings analyzed, 30.8% E3FAD pairings (88/286) resulted in no litter compared to 15.2% for E4FAD pairings (35/230) (χ^2^ = 16.98, p < 0.001) (Table 2).

### Fertility in E3FAD− and E4FAD− mice (non-carriers)

**Figure 3:**
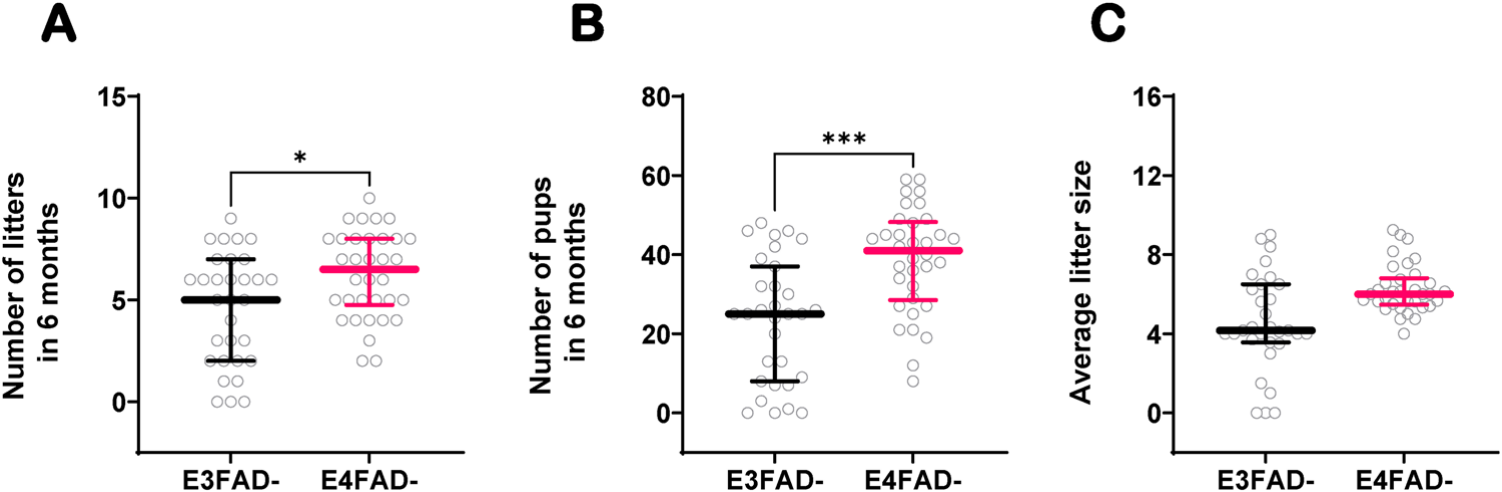
Fertility in colony III (EFAD−). Number of litters (**A**) and number of pups (**B**) produced by E3FAD**−** (n = 31) and E4FAD− (n = 34) breeding cages over a period of 6 months. Average litter size per breeding cage (**C**). Data is represented as scatterplots with median ± IQR. The differences between each group were determined by Mann–Whitney rank sum test. *p ≤ 0.05, ***p ≤ 0.001. In this colony, each breeding cage consists of trios of two females paired continuously with one male over the 6-month period.

Due to the presence of the 5xFAD mutations in the EFAD mouse model, we sought to verify that the genotype-specific differences observed in colony I and II were not due to an artifact caused by the presence of the transgenes. To test this, we assessed reproductive performance in colony III; a colony of EFAD− mice. Using the same measures as in colony I, we compared reproductive performance between E3FAD− and E4FAD− mice, carrying h-*APOE3* and h-*APOE4* respectively but no AD-associated transgenes. Our results show that the effect difference seen between E3FAD and E4FAD breeders was conserved in EFAD− mice. Notably, E3FAD− cages had significantly lower reproductive output over a period of 6 months, with 5.0 (2.0–7.0) litters and 25.0 (8.0–37.0) pups, compared to E4FAD− breeders which had 6.5 (4.8–8.0) litters (p = 0.019) and 41.0 (28.5–48.3) pups (p = 0.0002) (Fig. 3A,B). Additionally, the average litter size from E3FAD breeding cages was 4.2 (3.6–6.5) compared to 6.0 (5.5–6.8) in E4FAD females (p = 0.001) (Fig. 3C). Finally, E3FAD breeding cages in colony III had more non-productive cages than E4FAD cages (9.6% or 3/31 vs 0.0% or 0/34 respectively; p = 0.103) (Table 2).

### Gestational outcomes in E3FAD and E4FAD mice

**Figure 4:**
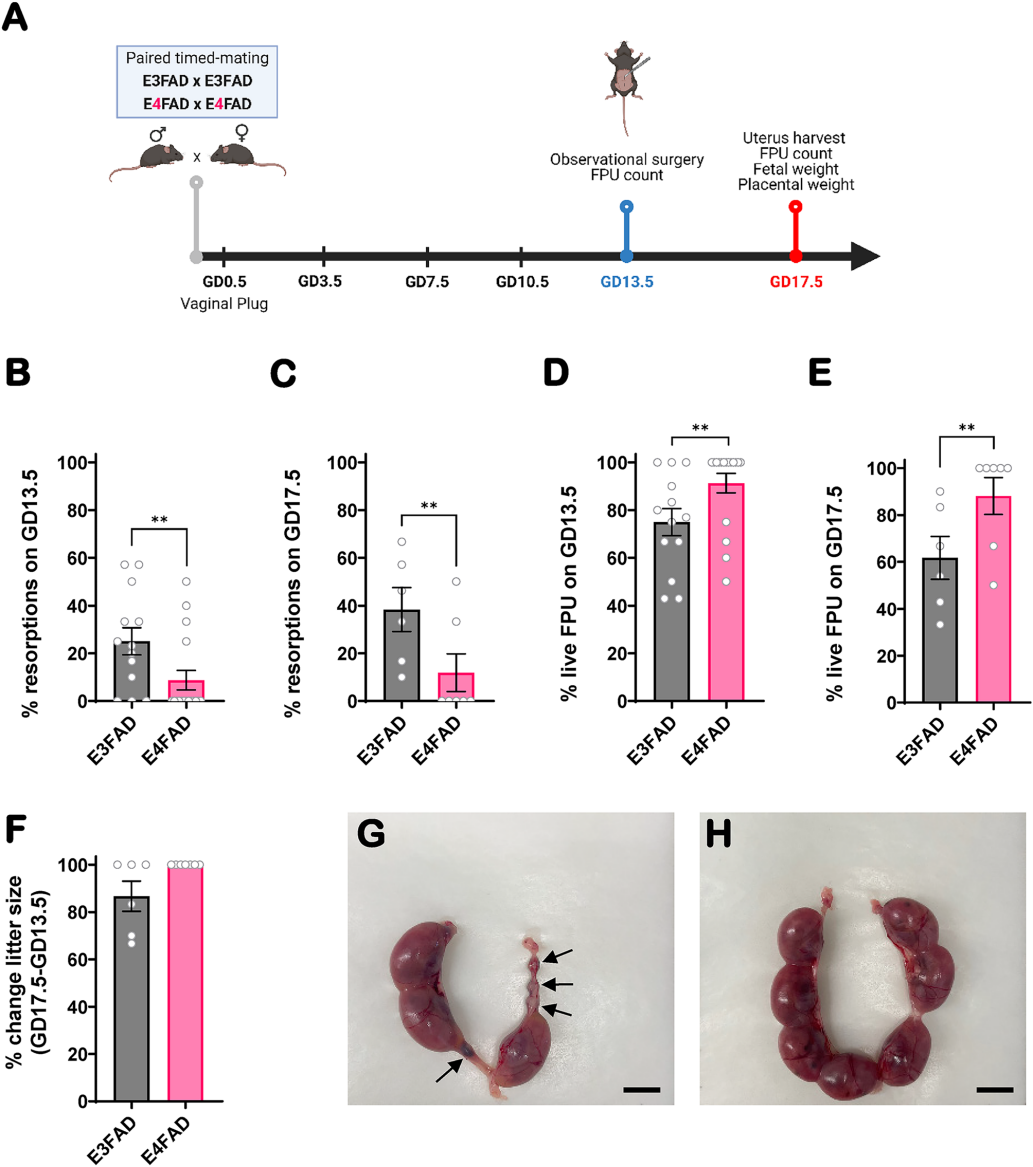
Pregnancy outcomes in EFAD mice. Diagram of gestation timeline, observational surgery, and experimental endpoints (**A**). Proportion of resorptions in E3FAD and E4FAD mice on GD13.5 (n = 13 and n = 17, respectively) (**B**) and GD17.5 (n = 6 and n = 7, respectively) (**C**). Corresponding proportion of viable fetal-placental units (FPU) at GD13.5 (**D**) and GD17.5 (**E**). % change in litter size between the day of surgery (GD13.5) and experimental endpoint (GD17.5) (**F**). Representative images of uteri harvested on GD17.5 from E3FAD (**G**) and E4FAD (**H**) pregnancies showing the presence of resorbed FPU (black arrow) in E3FAD (Scale bar: 1 cm). Data was tested for normality and represented as mean ± SEM (**B**–**F**). The differences between genotypes were determined by Chi-square test (**B**–**E**) or two-way ANOVA (**F**). **p ≤ 0.01. All mice used in this experiment were obtained from colony I.

To further explore fertility differences between *APOE3* and *APOE4* genotype, we examined gestational outcomes in E3FAD and E4FAD mice from colony I by counting the total number of viable fetal placental units (FPU) at gestational day (GD) 13.5 and GD17.5 in pregnant females of each genotype. A diagram describing our experimental approach is shown in Fig. 4A. Upon assessing fetal viability on GD13.5 and GD17.5 of gestation, we found that uteri from E3FAD dams had an increased proportion of fetal resorptions compared to E4FAD dams at both time points (Fig. 4B,C). On GD13.5, there were 25% resorbed FPUs in E3FAD compared to 8.7% in E4FAD (p = 0.006) (Fig. 4B). This trend was maintained on GD17.5 with 38% resorbed FPUs in E3FAD compared to 11.9% in E4FAD (p = 0.002) (Fig. 4C). This increased ratio of resorbed FPU corresponded to a reduced proportion of viable fetuses in E3FAD pregnancies at both GD13.5 and GD17.5 (Fig. 4D,E). To determine whether abdominal surgery had any effect on litter size, thus acting as a potential confounder of our results, we determined the percent change between the number of live fetuses recorded intra-operatively on GD13.5 and the number of live fetuses recorded on GD17.5 in both E3FAD and E4FAD mice. There was no significant difference in litter size between the start of the experiment and the experimental endpoint, suggesting that any potential effect of abdominal surgeries on litter size was not significantly different between the two genotypes (Fig. 4F, p = 0.250). Pictures taken on GD17.5 of gestation show the appearance of pregnant uterus from typical E3FAD and E4FAD dams (Fig. 4G,H). While four out of seven (57%) of the FPU were resorbed in the depicted E3FAD pregnancy (Fig. 4G) at GD17.5, no resorptions were noted in the E4FAD pregnancy (Fig. 4H).

**Figure 5:**
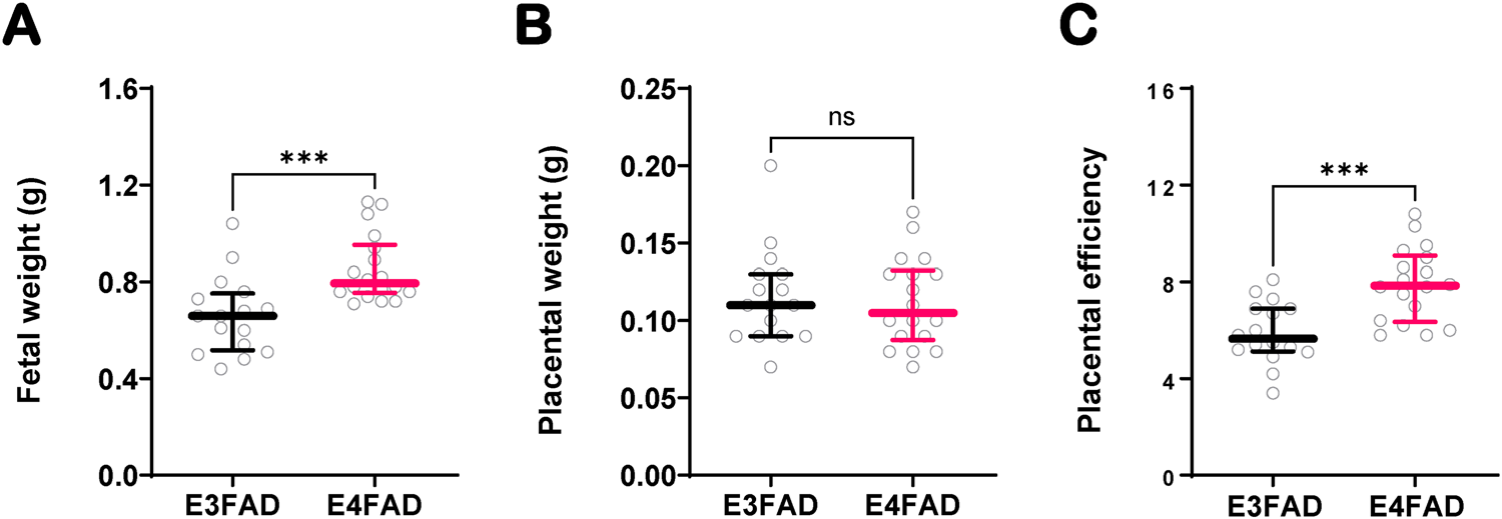
Fetal and placental outcomes in EFAD mice. Weights were recorded on for E3FAD (16) and E4FAD (n = 18) fetuses on GD17.5 and fetal weights were compared between genotypes (**A**). Placental weights were recorded concurrently (**B**), and placental efficiency was calculated as the ratio of fetal weight over placental weight for each FPU (**C**). Data is represented as median ± IQR and differences between genotypes were determined by Mann Whitney rank sum test for all comparisons. ***p ≤ 0.001. All mice used in this experiment were obtained from colony I.

We also measured weights for all viable fetuses on GD17.5. Fetal weight comparison between genotypes showed that E3FAD had a significantly lower weight than E4FAD fetuses (0.66 g vs. 0.80 g, p = 0.0003) at the same gestational age (Fig. 5A). Placental weights did not differ between genotypes, however, placental efficiency, a ratio of fetal weight per placental weight, was significantly reduced in E3FAD pregnancies compared to E4FAD (5.65 vs. 7.85, p = 0.0002) (Fig. 5B,C).

## Discussion

In this study, we report that *APOE4* is associated with increased fertility in two transgenic mouse models expressing h-*APOE*. We show that fertility and reproductive performance, as defined by number of litters, number of pups, and litter size, is increased in *APOE4* genotype relative to *APOE3* and *APOE2* genotypes. Intra-gestational examination of pregnancies in EFAD mice revealed that E3FAD mice have poorer reproductive health than E4FAD mice, as demonstrated by a higher proportion of resorbed FPUs, lower proportion of viable FPUs, lower fetal weights, and diminished placental efficiency. Taken together, these results provide further evidence that *APOE* polymorphisms may play an important role in female fertility and highlight APOE KI mice as an effective tool to study genotype-specific effects of *APOE* on reproductive physiology.

To date, only a few studies have examined the potential effect of *APOE* genetic polymorphisms on human fertility and reproduction^5,8,9,19–23^. Of these studies, two examined the impact of genotype on male fertility^21,23^, another one assessed fertility in both men and women at post-reproductive age^22^, and a total of four studies investigated the role of genotype on female fertility^5,8,9,19^. In the studies that investigated fertility in males, *APOE3/4* genotype was associated with lower fertility than *APOE3/3* genotype as assessed by the number of children per individual males and an increased odds ratio of infertility in *APOE4* male carriers. In a study by Corbo et al., *APOE4* female carriers were also shown to have lower fertility compared to *APOE3/3* females^22^. However, to date, most other studies that investigated female fertility report a dissimilar trend. In these studies, the highest reproductive performance was observed in *APOE4* carriers, a trend that is concordant with our findings in transgenic mice. Also, in line with our findings, all human studies in which the allele was present attributed the lowest reproductive efficiency in females to *APOE2* genotype. Interestingly, despite varying housing and husbandry conditions between colonies, we found that the association between genotype and fertility was maintained in all three colonies. Notably, the lengthened 14-h light cycle in colony II compared to the 12-h cycle in colonies I and III did not affect the *APOE4*-associated phenotype despite the well-known influence of light on circadian rhythms and mammalian fertility^24,25^. Studies have shown that extending the time of light exposure by 2 h from 12 h (12:12 light–dark cycle) to 14 h as is the case in the 14:10 cycle induces more regular estrous cycles and shortens cycle length resulting in improved reproductive outcomes^25,26^. This phenomenon may influence the results seen in colony II and should be taken into account when interpreting findings. The differences in litter size found in our model were not evident in a study by Holden et al.^27^. In this study, the authors reported the total number of litters, offspring, and average litter size in APOE-TR mice and human APP KI mice NL-G-F and NL-F breeding pairs; each strain expressing human *APOE2*, *APOE3*, or *APOE4*. Average litter sizes did not differ between NL-G-F/APOE3 and NL-G-F/APOE4 mice, contrary to our findings in EFAD mice. The same observation applied to mice of NL-F background. EFAD mice express 5 AD-related transgenes which include Swedish (K670N/M671L), Florida (I716V), and London (V717I) mutations in APP, and M146L and L286V in PSEN1. In contrast, NL-G-F mice express 3 different AD-related transgenes in APP (Swedish [K670N/M671L], Iberian [I716F], and Artic [E693G]) and NL-F only express the Swedish and Iberian mutations^28,29^. This may suggest that the aforementioned mutations in human APP are not sufficient to observe differences in fertility. Moreover, the average litter sizes in the study by Holden et al. were determined on a small sample size of 1–3 breeding pairs for each genotype and no statistical analysis was available. Thus, these results cannot be extrapolated to other APP KI mouse models. Because our data is obtained from homozygous crosses, we are unable to address the contribution of male genotype to the reported genotype-associated reproductive outcomes. However, the ability to reproduce *APOE* genotype effects on female fertility in EFAD mice, opens the door for the possibility of investigating paternal contributions to reproductive health and output through carefully designed heterozygous matings.

Importantly, there are robust differences in both the distribution of *APOE* alleles and their effect across human populations, which adds complexity to interpreting *APOE* genotype effects on fertility. For instance, the only study that reported an association between *APOE3/3* genotype and higher fertility in female was done in Southern Italy, while the other studies which reported *APOE4* as the allele associated with the highest fertility were done in natural fertility populations of Ecuador, Bolivia, and Ghana^5,8,9,22^. Natural fertility populations are characterized by their isolation from modern world influences, hence the environment those populations live in, and their lifestyle vary grossly. These different environmental pressures are strongly associated with *APOE* genotype distribution. For example, the prevalence of *APOE4* is 9.1% in Mediterranean European populations living in Southern Italy, compared to considerably higher frequencies of 28.0–28.9% in the African–Ecuadorian and Cayapa Indians of Ecuador, 20% in the indigenous Tsimane of Bolivia, and 14.9% in the Ghanaian population^5,7–9^. The high variance in allelic distribution suggests important gene-environment interactions that impose different selective pressures. Factors such as diet, geographic location (including temperature, altitude, or latitude), and infectious burden have been described as some of the selective forces that have influenced the prevalence of one *APOE* variant over the other^2,30^. For instance, higher prevalence of *APOE4* in some native populations of South America and West Africa is thought to be an adaptive mechanism in regions that have a high pathogenic burden^5,31^. In such an environment, *APOE4*’s pro-inflammatory properties may confer a protective advantage against severe infections and subsequent premature death. Consistent with this putative protective effect, Oriá et al. showed that *APOE4* was correlated with a lower diarrhea burden in Brazilian children living in an environment with high exposure to enteric parasites^32,33^. Going one step further, van Exel et al. looked at the relationships between *APOE4*, pathogenic burden and fertility and found that *APOE4* was associated with higher fertility in a subset of women exposed to high pathogen levels^5^. Along with its benefits for fertility, *APOE4* also was associated with improved cognitive function in populations with a high parasite burden^32,34^. These findings suggest that carrying the *APOE4* allele in adverse environmental conditions provides better chances of survival through the earlier periods of life. Investigating similar interactions in EFAD mice and other *APOE* KI mouse models will allow for better control of the environmental influences that otherwise complicate the study of *APOE*-associated fertility in humans.

Despite some positive effects on survival, *APOE4* remains detrimental in post-reproductive life stages. Even though the gene carries a fertility advantage, fertility does not appear to be linearly associated with increased lifespan. In 1977, Kirkwood and Rose formulated the “disposable soma theory”, which implies that fertility and improved reproductive capacity come at the cost of late survival and longevity^35^. This trade-off would be due to the increased metabolic cost, risk of disease, and risk of mortality associated with reproduction^36,37^. Numerous human and animal studies have established a link between early-life reproduction and increased risk of mortality despite enhanced survival benefits^37–42^. Thus, it is not surprising that as living conditions evolved natural selection may have shifted toward a version of the gene that improved longevity. To modern-day human populations, pathogens constitute less of a threat and food availability has improved as humans moved from foraging times to agricultural and industrialized civilization. Given the trend we see in fertility (E4 > E3 > E2), our findings also support that *APOE3* and *APOE2* may have indeed selected through evolution at the cost of fertility^43,44^. Furthermore, it has been established that the *APOE4* allele is the parent allele from which *APOE3* and *APOE2* derived from in chronological order^43,45^. Indeed, out of the three alleles, *APOE4*’s gene sequence resembles the non-polymorphic *APOE* gene carried by other animal species the most, including the great apes species from which humans evolved^45^. Nevertheless, while there is a high degree of homology, key differences remain between h*-APOE* and its preceding counterparts. For instance, h*-APOE* has an arginine at position 61, while most other species, including mouse, have a threonine; a unique change that contributes to h-*APOE* lipoprotein binding preferences^46^. This raises a question about what other functional domains outside of the common polymorphisms may impact APOE’s effects on reproduction.

The observed reduction in fecundity seen with carriage of *APOE3* and *APOE2* may be related to APOE’s main physiological function, which is the transport and regulation of cholesterol and other lipids. The *APOE* alleles, which result from a unique combination of two single nucleotide polymorphism (SNP) sites (rs429358 and rs7412), lead to three isoforms with significant structural differences (APOE2: Cys^112^/Cys^158^, APOE3: Cys^112^/Arg^158^, APOE4: Arg^112^/Arg^158^)^47,48^. The amino acid changes that characterize each isoform have a drastic effect on cholesterol metabolism by causing variations in their lipid binding capacity. Indeed, while APOE4 preferentially binds to triglyceride rich very-low-density lipoproteins (VLDL), APOE3 and APOE2 bind to high-density lipoproteins (HDL)^48–50^. With this shift, APOE3 and APOE2 are associated with lower circulating levels of triglycerides and cholesterol than APOE4^49–51^. Because cholesterol is essential for fetal development and is the precursor for biosynthesis of progesterone and estrogen, the decrease in cholesterol bioavailability may correlate with the poorer reproductive outcomes associated with APOE3 and APOE2. Supporting this potential relationship between *APOE* genotype and steroidogenesis, APOE is synthesized by ovarian granulosa cells and is hypothesized to participate in cholesterol intake by both granulosa cells which synthesize estradiol, and theca cells which synthesize progesterone^20^. Moreover, Jasienska et al. showed that women who are *APOE4* carriers have approximately 20% higher levels of circulating progesterone during their menstrual cycle than non-carriers^19^.

The mechanisms driving the effect of *APOE* genotype on lipid metabolism and steroidogenesis are not well understood but could also explain the decreased fetal viability and low fetal weight we see in E3FAD mice. While there is evidence supporting the association between APOE3 genotype and increased fetal loss in humans^8^, previous reports from a meta-analysis argues that *APOE3* genotype is protective against recurrent pregnancy loss while *APOE4* could be a risk factor^52^. While we see lower weight in fetuses of *APOE3* genotype, an association between APOE genotype and fetal growth restriction, a pathology that is characterized by lower fetal weight, has not been reported^53,54^. Placental efficiency, a measure of the placenta’s ability to support fetal growth, had never been investigated in EFAD mice. We report for the first time that, at baseline, E3FAD mice have a lower placental efficiency than E4FAD mice; an observation which supports a reduced reproductive potential with *APOE3* genotype. Finally, *APOE4* has been associated with an earlier age at first reproduction^8^ but this finding is not reproduced in our study by measuring length of pairing before birth. Important confounders that may have masked this effect in our study include the use of breeding trios and investigator-controlled timing of pairings that do not allow us to account for effects on individual females.

Overall, despite what seem to be worse reproductive outcomes with *APOE3*, the variant has gradually and effectively replaced its ancestral counterpart as the more predominant allele in humans. This has been attributed to the improved suitability to the evolving lifestyle of human populations and positive association with longevity^30,55^. While the EFAD mouse model was originally developed for the study of aging and sex-related related effects of APOE on AD, it has now proven to be an effective tool to study the complex relationship between *APOE* genotype and reproductive health. Yet, the current findings prompt us to re-examine our views on the association between *APOE3* and normality in the context of reproductive physiology. Further research needs to be pursued to elucidate the isoform’s role in the regulation of pregnancy hormones biosynthesis and the specific mechanisms driving gene-environment interplay. Particularly, experiments that include heterozygous matings should be performed as they would be more representative of the natural distribution of genotypes in populations.

Previous literature suggests a greater risk of AD in women with ≥ 5 pregnancies^56^. Having children has also been associated with an increased risk of receiving an AD diagnosis later in life although a link between AD risk and the number of children was not found^57^. Additionally, a study by Gilsanz et al. concluded that a shorter reproductive (fertile) window was associated with increased AD risk^58^. Assuming that each woman has a baseline risk for AD influenced by APOE genotype and history of having children, it is not known if having a pregnancy complicated by preeclampsia or preterm labor further accelerates the underlying pathophysiologic processes leading to AD. Modeling pregnancy complications in EFAD mice could help with these endeavors. If true, there is a population of women who could benefit from AD screening and early therapeutic interventions in the future. Conversely, and as a more immediate measure, clinical trials of therapeutic intervention aimed at optimizing pregnancy outcomes should consider accounting for *APOE* genotypes in subgroup analyses.

## Methods

### Experimental design

Breeding records from three distinct animal colonies (colony I, colony II, and colony III) were retrospectively analyzed to establish fertility and reproductive outcomes in two transgenic mouse models carrying h-*APOE*. Detailed characteristics including location, principal investigator, and genetic background for each colony are described in Table 1. To supplement reproductive output data obtained from breeding records, we used an observational approach to assess the effects of *APOE* genotype on pregnancy.

### Animal colonies

The animal colonies included in this study were selected based on the genetic background of the mice in each colony. As detailed in Table 1, colony I refers to the original EFAD mouse colony housed in the University of Illinois at Chicago (UIC) College of Medicine. This transgenic mouse model was originally developed by Dr. Mary Jo LaDu as previously described^12^. In brief, EFAD mice were developed by crossing APOE-targeted replacement (APOE-TR) mice with 5xFAD mice. APOE-TR mice are a KI mouse model developed by gene replacement of mouse *APOE* with each of the h-*APOE* alleles, allowing for physiological expression of h-*APOE* under the control of the endogenous mouse *APOE* promoter^11,59–63^. As a result of the targeted replacement strategy, these mice do not express murine *APOE*^59–61^. 5xFAD mice express five familial AD-associated (5xFAD) mutations [APP K670N/M671L + I716V + V717I and PS1 M146L + L286V] under the control of the thy-1 promoter. Hence, EFAD mice are 5xFAD^+/−^/h-*APOE*^+/+^ and EFAD− mice, which do not carry the 5xFAD transgenes, are 5xFAD^−/−^/h-*APOE*^+/+^^13^. The colony is maintained by mating two 5xFAD carrier females (5xFAD^+/−^/h-*APOE*^+/+^) with one 5xFAD non-carrier males (5xFAD^−/−^/h-*APOE*^+/+^) (at 2–3 months of age) and breeding cages (breeding trios) were maintained continuously until retirement after 6 months of breeding. The average age of female breeders at the time of initial mating was of 9.2 weeks for E2FAD, 9.1 weeks for E3FAD, and 8.4 weeks for E4FAD mice (Table 1). Mice are maintained on a 14 h light/10 h dark light cycle in a room set at 75°F and 50% room humidity. Animals have ad libitum access to standard irradiated rodent chow (irradiated LM-485 Mouse/Rat Diet, Teklad, Cat#7912) and autoclaved tap water. Following weaning, all mice are ear tagged with a unique ID number, and a tissue sample (tail snip) is collected for determination of 5xFAD genotype, and confirmation of *APOE* genotype. All mice are monitored at least daily by lab staff and/or UIC Biologic Resources Laboratory (BRL) care staff.

Colony II is a second EFAD colony housed at the University of Southern California (USC) by Dr. Christian Pike. This colony was started using EFAD mice transferred from colony I in 2018. Colony II has a different breeding scheme compared to colony I. Mice in this colony are maintained on a 12 h light/12 h dark light cycle and are bred by mating one 5xFAD carrier female (5xFAD^+/−^/APOE^+/+^) with a 5xFAD non-carrier male (5xFAD^−/−^/APOE^+/+^). In this colony, the average ages of female breeders at the time of initial mating were 11.4 weeks for E3FAD and 13.2 weeks for E4FAD females (Table 1). Pairs have ad libitum access to food (irradiated PicoLab Rodent Diet 20 formulation, Lab Diet, Cat#5053) and water and are monitored by lab staff and USC veterinarians. Females are checked for pregnancy weekly. If a female is seen to be pregnant, she is separated from the male and housed in a new clean cage with extra nesting material. The cage is not changed until pups are 5–7 days old. After pups are weaned at 22–23 days, females are usually paired with a different male and the process is restarted. If no suitable male is available, the female is housed without a mate until an appropriate partner exists or, if necessary, is re-paired with the same male. Creating the same breeding pair is avoided whenever possible. Females are usually retired after birthing 3 litters or when they are 8–10 months of age, whichever comes first.

Colony III represents a colony of EFAD− mice also housed at UIC and maintained by Dr. Leon Tai. Mice in this colony are maintained under the same housing and husbandry conditions, as well as receive the same diet as mice colony I. Mice in this colony are bred by mating two EFAD− females with an EFAD− male in breeding trios for a continuous period. The average age of female breeders at the time of mating was of 7.4 weeks and 6.7 weeks for E3FAD− and E4FAD− females respectively (Table 1).

Breeder mice were maintained on the same diet as non-breeders in all three colonies. All experiments were approved by the Institutional Animal Care and Use Committee at the University of Illinois at Chicago (colony I and colony III) or the Institutional Animal Care and Use Committee at the University of Southern California (colony II) in accordance with National Institute of Health standards. We confirm this study is reported in accordance with the ARRIVE (Animal Research: Reporting of In Vivo Experiments) guidelines as outlined at https://arriveguidelines.org.

### Breeding records analysis

For colony I, we retrospectively analyzed four years of breeding records spanning breeding trios started between July 2016 and November 2020. The records included detailed information such as date of birth and age of breeding females at the time of trio setup, date of parturition for each litter produced, and number of pups for each litter. For each cage, a cutoff was set at 180 days post trio setup to delineate a 6-month breeding period. Within that period, we extracted the total number of litter and pups produced per breeding cage. The average litter size for each breeding cage was determined by dividing the total number of pups produced by total number of litters produced for each cage.

For colony II, records from March 2018 to July 2023 were analyzed. From these records, we calculated the length of pairing before birth of litter for each pair as the interval between the date each pairing was started and the date of birth of each litter. The number of pups weaned after each productive pairing was used to describe litter size.

For Colony III, we analyzed one year of breeding records spanning July 2018 to August 2019. Analysis was performed similarly to Colony I.

### Pregnancy phenotyping

To explore genotype-specific effect on pregnancy, we used an observational approach to visualize and describe reproductive outcomes in the pregnant female on GD13.5 and GD17.5. These mice originated from colony I and targeted experiments were performed from 2021–2023. To initiate pregnancy, a 5xFAD carrier female (5xFAD^+/−^/h-*APOE*^+/+^) was placed with a 5xFAD non-carrier male (5xFAD^−/−^/h-*APOE*^+/+^) overnight. The next day, females were separated from the male cage and the presence of a gestational plug was marked as GD0.5. The presence of a visually enlarged abdomen and a minimum maternal weight gain of 2.0 g between GD7.5 and GD12.5 were used to confirm pregnancy. On GD13.5, pregnant females were anesthetized with 2% isoflurane. A mid abdominal incision was made and the skin and abdominal muscle layers were gently pulled to expose the uterus. Only one member of the research team (BMF) performed all the surgeries. The number of implantation sites and numbers of live and dead or resorbed fetuses were counted through the uterine wall. The abdominal incision was then closed using a two-layer suture method. Post-operatively, the females were single housed in a clean cage and monitored closely until GD17.5. On GD17.5, experimental females were sacrificed, and the uterus was exteriorized for a final count of live and dead fetuses. Fetuses and their matching placentas were gently taken out from the uterus and weighted. The ratio of fetal weight to placental weight was calculated to assess placental efficiency. A diagram representing the timeline of these experiments was created using BioRender.com.

### Statistical analysis

Statistical analysis was performed using GraphPad Prism version 10.0.2 and SigmaPlot (Systat, version 15.1). Results are presented as median ± interquartile range or mean ± SEM as appropriate. Differences between genotypes were assessed using Mann–Whitney rank sum test when comparing two groups. Kruskal Wallis ANOVA on ranks with Dunn’s post-hoc analysis was used to assess differences between groups with uneven sample sizes. Proportions were compared using Chi-square (χ^2^) or Fisher Exact Test if over 20% of the expected values were less than 5. A two-tailed *p* value equal to or less than 0.05 was considered statistically significant in all analyses.

## Data Availability

The data that support the findings of this study are available upon written request to the corresponding author.
